# Price Forecasting of Marine Fish Based on Weight Allocation Intelligent Combinatorial Modelling

**DOI:** 10.3390/foods13081202

**Published:** 2024-04-15

**Authors:** Daqing Wu, Binfeng Lu, Zinuo Xu

**Affiliations:** 1 College of Economics and Management, Shanghai Ocean University, Shanghai 201306, China; 2 China Fisheries Development Strategy Research Center, Shanghai 201306, China; 3 School of AI and Advanced Computing, Xi’an Jiaotong-Liverpool University, Suzhou 215400, China

**Keywords:** price forecasting, marine fish, intelligent combinatorial modelling, neural network

## Abstract

China is a major player in the marine fish trade. The price prediction of marine fish is of great significance to socio-economic development and the fisheries industry. However, due to the complexity and uncertainty of the marine fish market, traditional forecasting methods often struggle to accurately predict price fluctuations. Therefore, this study adopts an intelligent combination model to enhance the accuracy of food product price prediction. Firstly, three decomposition methods, namely empirical wavelet transform, singular spectrum analysis, and variational mode decomposition, are applied to decompose complex original price series. Secondly, a combination of bidirectional long short-term memory artificial neural network, extreme learning machine, and exponential smoothing prediction methods are applied to the decomposed results for cross-prediction. Subsequently, the predicted results are input into the PSO–CS intelligence algorithm for weight allocation and to generate combined prediction results. Empirical analysis is conducted using data illustrating the daily sea purchase price of larimichthys crocea in Ningde City, Fujian Province, China. The combination prediction accuracy with PSO–CS weight allocation is found to be higher than that of single model predictions, yielding superior results. With the implementation of weight allocation intelligent combinatorial modelling, the prediction of marine fish prices demonstrates higher accuracy and stability, enabling better adaptation to market changes and price fluctuations.

## 1. Introduction

Given rapid economic development and the increase in disposable income in recent decades, consumption structure has been in a state of constant upgrade, with residents placing greater emphasis on dietary health and protein intake. As a rich source of protein, marine fish play a significant role in the daily diets of people worldwide and hold an important position globally. The evolving consumer mindset has led to a significant increase in the demand for marine fish [1]. However, as the demand for healthy diets continues to rise, the volatility of marine fish prices has also attracted widespread attention.

In recent years, there have been frequent fluctuations in marine fish prices, which has had a severe impact on people’s lives and national economic stability. For instance, in the Ningde area of the Fujian Province, China, there have been substantial price fluctuations in artificially farmed larimichthys crocea. In the period leading up to the Chinese Spring Festival in 2020, fish farmers urgently sold off large quantities of larimichthys crocea to recoup their investments, resulting in a significant increase in supply and a subsequent depression in prices, which fell below 3.15 US dollars/kg. Before the Mid-Autumn Festival, the supply was severely inadequate, leading to a substantial increase in acquisition prices, reaching over 5.83 US dollars/kg [2]. The price volatility of marine fish affects the decision-making processes of producers, consumers, governments, and other stakeholders. Therefore, accurately predicting changes in marine fish prices has become an urgent need for decision-makers and relevant stakeholders. Establishing an efficient and accurate marine fish price forecasting model is crucial in preventing adverse impacts on people’s lives caused by unforeseen events. This measure plays a significant role in addressing issues related to agriculture, rural areas, and farmers, and in promoting agricultural informatization. The concept of “food systems thinking” emphasizes the importance of healthy diets and the complexity of relationships between foods. In this study, we will apply this concept, integrating marine fish price forecasting into a broader food system [3]. Through this approach, we aim to establish a predictive system for marine fish prices, providing decision-makers with more accurate information and promoting sustainable development and precise agricultural management.

Predicting prices of marine fish is a focal issue in the field of marine fish price research, and research methods exhibit diversification. The price of marine fish demonstrates complex volatility and nonlinear characteristics, representing a typical complex time series that poses significant challenges for accurate forecasting. Traditional econometric methods commonly employed in price forecasting include autoregressive integrated moving average (ARIMA) [4] and exponential smoothing (ETS) [5]. These methods have been widely used in the field of price forecasting, where they have been adapted to various agricultural product price characteristics, gradually improving the predictive capacity of models as historical data becomes more abundant and precise [6,7]. Machine learning (ML) methods possess powerful data-driven attributes and adaptive learning capabilities, enabling them to effectively extract hidden factors that traditional methods fail to capture. Models such as extreme learning machine (ELM) [8] and long short-term memory (LSTM) networks [9] are employed to output results. Compared to traditional econometric methods, these models exhibit higher accuracy, robustness, and generalization, allowing for more precise prediction of agricultural product prices. On one hand, when dealing with high-dimensional and large-scale prediction problems, shallow machine learning algorithms like support vector machines (SVM) and backpropagation neural networks (BPNN) face significant limitations, including the curse of dimensionality and ineffective feature representation [10]. On the other hand, although individual model prediction errors fluctuate greatly, overall precision decreases as the prediction horizon lengthens. However, not all artificial intelligence models outperform traditional econometric forecasting methods in practical predictions [11,12]. In previous research on predicting the prices of aquatic products, Hasan et al. [13] used the ARIMAX model to forecast catfish prices, and the results indicated that the model has high predictive accuracy both in-sample and out-of-sample. Nam and Sim [14] improved the accuracy of the ARIMA model by using the improved Diebold Mariano test, demonstrating better performance in predicting abalone prices. Gordon [15] used the ARDL/Bounds model to forecast lobster prices. Wu et al. [16] used the VMD-IBES-LSTM mixed method to predict the prices of five aquatic products in China, including grass carp, crucian carp, carp, silver carp, and scallops. The results showed that this method better explained the seasonality and trend of changes in the consumer price index of aquatic products in China. Hence, appropriate prediction models should be selected based on the characteristics of the data and task at hand.

Combination models, by integrating the advantages of traditional statistical methods, intelligent optimization algorithms, and artificial intelligence techniques, set prior assumptions and perform data processing for prediction problems, thereby reducing learning biases and significantly enhancing the fitting ability of predictive models [17]. In terms of research methodology, scholars have gradually developed a “decompose–integrate” hybrid model, which has improved predictive performance to some extent. Unlike general hybrid models, the decompose–integrate framework first decomposes agricultural product prices into multiple components, and then predicts each component using corresponding forecasting methods. Since its inception, this approach has been applied in various fields, such as commodity prices and energy, yielding favorable results [18]. In the field of complex time series forecasting modeling, the decompose–integrate methodology is considered an effective strategy for improving prediction accuracy. Its core idea is to use signal decomposition algorithms to break down complex time series into a series of relatively simple and stationary sub-sequences, thereby reducing the complexity of prediction modeling tasks [19]. Techniques such as empirical wavelet transform (EWT), empirical mode decomposition (EMD) [20], and singular spectrum analysis (SSA) [21] have been adopted to decompose the original data sequence and eliminate noise in the time series. By separately modeling the decomposed components, such as trend, seasonality, and residuals, and recombining them to obtain the predicted values of the original time series, more accurate forecasting results can be achieved compared to directly modeling the original time series.

However, a substantial amount of theory and practice have demonstrated that it is impossible for a single model to capture both linear and nonlinear patterns in agricultural product price sequences. Therefore, scholars have introduced hybrid models for price prediction. A hybrid model combines the prediction results of different forecasting methods to form new predictions. The most used combination strategy is to use statistical methods, such as the mean method, median method, minimum error method, and more complex Bayesian averaging method, to determine weights [22]. This strategy achieves complementarity among individual models, thereby capturing the underlying patterns in the sequence more accurately [23]. This conclusion has been proven in previous time series literature [24]. In the field of swarm intelligence optimization algorithms, research has shown that the cuckoo search (CS) algorithm has stronger comprehensive advantages in terms of parameter number, versatility, and global optimization ability, and can flexibly combine with other algorithms such as PSO, demonstrating wider applicability [25].

The research findings presented above indicate that time series decomposition exhibits high accuracy and wide applicability in predicting marine fish prices. However, single-model predictions have disadvantages such as limited adaptability, inadequate precision, and poor robustness. Building upon previous research, this study proposes a weight allocation method based on particle swarm optimization with cuckoo search algorithm (PSO–CS). This paper presents several novel contributions:

(1) We introduce a weight allocation intelligent combinatorial modelling forecasting framework, which strategically assigns weight distribution across distinct models. This framework exhibits marked superiority in terms of flexibility, precision, and robustness.
(2) In our approach, prior to forecasting, we employ various decomposition techniques to partition the original dataset into multiple sub-series. This method accentuates intricate details within the time series, thereby rendering sub-series fluctuations smoother relative to the initial series, and subsequently improving predictive accuracy.
(3) We leverage the innate capabilities of self-learning and social learning introduced by the PSO–CS algorithm to enable an enhancement in global search efficiency.

The remainder of this paper is organized as follows. Section 2 briefly introduces the construction of the PSO–CS algorithm. Section 3 presents a weight allocation intelligent combinatorial modelling forecasting framework. Section 4 provides an overview of the data context. In Section 5, the results of the experiments are analyzed. Finally, the conclusion is given in Section 6.

## 2. Fish Price Prediction Framework Based on PSO–CS Weight Allocation Algorithm

The basic idea of the particle swarm iteration-based cuckoo hybrid search optimization algorithm is in the iterative process of particle swarms. The PSO algorithm is used to update the velocity and position of the particles in each generation to obtain the optimal position of a group of particles, and then the optimal particle position is entered into the CS algorithm to continue to iteratively update. Based on the number of iterations of the original algorithm, each particle swarm adds one update and calculation of the CS algorithm, and there is little change in the running time.

Given m individual forecasting models, each assigned a corresponding weight denoted as $w_{i}\left(1,2,\ldots ,m\right)$, the combined forecast can be expressed as:

(1) $${\hat{y}}_{\mathrm{combined}}={\hat{y}}_{1}*w_{1}+{\hat{y}}_{2}*w_{2}+\cdots +{\hat{y}}_{m}*w_{m}$$

For optimal weight assignment of combined models, determining the weights of each individual prediction model is critical. In this study, the corresponding weights for each individual model are estimated by constructing an optimization problem that minimizes the error between the combined predicted and observed values.

Thus, an optimization problem estimating the reasonable weight of each individual model is construed as below:

(2) $$Min\;G\left(y-{\hat{y}}_{\mathrm{combined}}\right)\;s.t.\sum_{i=1}^{m}w_{i}=1$$

where G is a predetermined function, such as sum squared (SSE), mean squared (MSE), sum absolute (SAE), mean absolute (MAE), etc., and y is the observed value.

The proposed PSO–CS weight assignment method is based on an improved CS algorithm, namely the PSO–CS algorithm. The CS algorithm, inspired by cuckoo’s brood parasitic behavior, is a new population-based search paradigm. Cuckoos seek out nests of other hosts and then lay their eggs, which may be found and discarded by the hosts. To enhance the survival rate of their eggs, cuckoos can imitate the host’s eggs, or even take them out. The CS algorithm is efficient, robust, and relatively simple in comparison with other evolutionary computing algorithms due to its few control parameters. Furthermore, the levy flight, instead of standard random walk, is applied in CS. This helps to explore the huge solution space compared to the linear relationship, due to its infinite mean and variance, as well as its nonlinear relationship. However, the huge exploration space of the CS algorithm may lead to poor convergence and accuracy of solutions, so the PSO algorithm is introduced to optimize the CS algorithm. The proposed PSO–CS weight assignment method can be expressed as follows:

The objective function $F(w)=\sum_{i=1}^{n}y-\left|{\hat{y}}_{n}*x_{n}\right|$ is defined, where $x_{n}$ is the value of the weight coefficient, ${\hat{y}}_{n}$ is the nth individual forecasting model, and $\sum_{i=1}^{n}x_{i}=1$.

Parameters include population size N, maximum number of iterations T, minimum weighing value $w_{min}$, maximum weight $w_{max}$, the acceleration coefficients $c_{1}$ and $c_{2}$, maximum discovery, and probability $p_{a}\in [0,1]$. A set of randomly generated host nests $X_{i}=\left\{x_{i1},x_{i2},\ldots ,x_{iD}\right\}$ and the corresponding velocities $V_{i}=\left\{v_{i1},v_{i2},\ldots ,v_{iD}\right\}$, and host nests $X_{i}(i=1,2,\ldots ,N)$ to its location in the D-dimensional space, is a potential solution to the problem. In the t iteration, the update speed of the first i (the first nest) is built by optimizing the location of the first i.

(3) $${pbest}_{i}^{\left(t\right)}=\left\{{pbest}_{i1}^{\left(t\right)},\ldots ,{pbest}_{i,D}^{\left(t\right)}\right\}$$

The optimal position for the entire population $gbest^{\left(t\right)}$ is calculated, and then the nested positions are updated:

(4) $$v_{ij}^{\left(t+1\right)}=w\times v_{ij}^{\left(t\right)}+c_{1}\times \mathrm{rand}\left(0,1\right)\times \left[pbest_{ij}^{\left(t\right)}-x_{ij}^{\left(t\right)}\right]\;+c_{2}\times \mathrm{rand}\left(0,1\right)\times \left[gbest_{j}^{\left(t\right)}-x_{ij}^{\left(t\right)}\right]$$

(5) $$x_{ij}^{\left(t+1\right)}=x_{ij}^{\left(t\right)}+v_{ij}^{\left(t+1\right)}$$

These are $v_{i,j}^{\left(t+1\right)}$ and $x_{ij}^{\left(t+1\right)}$, and they represent (t+1) of $w=w_{max}-\frac{w_{max}-w_{min}}{T}\times t$, which are the inertia weights. $x_{ij}^{\left(t\right)}=pbest_{ij}^{\left(t\right)}$ updates the $x_{ij}^{\left(t+1\right)}$, where $L(\lambda )∼u=t^{-\lambda }(1<\lambda \leq 3)$ is $x_{ij}^{\left(t+1\right)}=x_{ij}^{\left(t\right)}+\alpha ⊛L(\lambda )$, and α is the step size, which should be proportional to the size of the optimization problem. This $x_{ij}^{\left(t+1\right)}$ will change randomly with probability $p_{a}$.

Replacements $x_{ij}^{\left(t+1\right)}$ are defined by the $x_{ij}^{\left(t\right)}$ if the fitness value $F\left(x_{ij}^{\left(t+1\right)}\right)>F\left(x_{ij}^{\left(t\right)}\right)$. Then, the list of nests is found by sorting the list of t generations of the best nests.

The PSO–CS hybrid optimization algorithm combines the search capability of PSO and the global search capability of CS to improve the optimization capability of the algorithm in general. Experimentally, it has been proven that the accuracy of the PSO–CS hybrid optimization algorithm is significantly better than that of the PSO algorithm, and it is more stable [26].

## 3. Forecasting Framework

**Figure 1:**
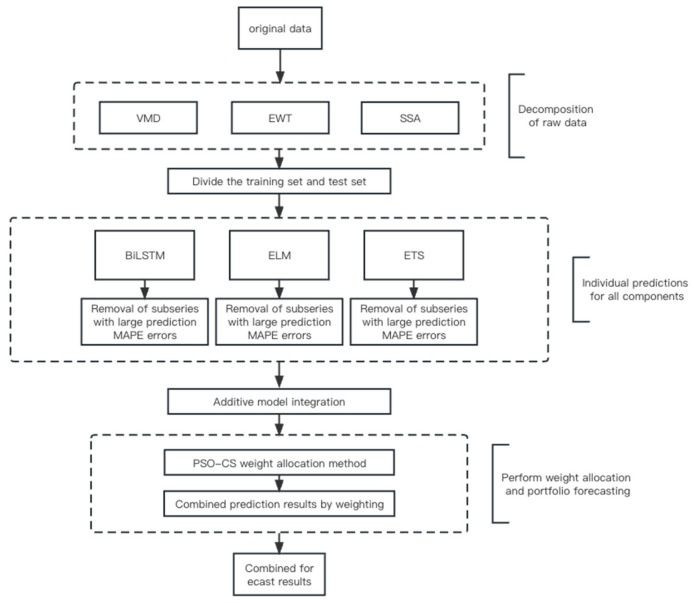
Diagram of the combined forecasting framework.

This article proposes an optimal combination framework for predicting the prices of marine fish based on the PSO–CS weighted aggregation model, as shown in Figure 1. The framework consists of five steps:

- Step 1: Data decomposition. The price data of marine fish is decomposed into multiple sub-sequences using EWT, VMD, and SSA decomposition techniques, aiming to identify the main sequences.
- Step 2: Individual prediction. The selected sub-sequence components are sequentially inputted into various prediction models, including LSTM, ELM, and ETS models, to obtain predictions for each component.
- Step 3: Removal of large errors. An error analysis is performed on the predicted results of the components. Components with significant errors are removed, and the original sequence of the removed component is merged with the residual sequence.
- Step 4: Combination. The predicted results of each group of components are summed to obtain the final prediction result for that group.
- Step 5: Prediction aggregation. The PSO–CS weight allocation method is employed to determine the optimal weights for different individual predictions. Then, the predictions of each component are weighted accordingly to obtain the final aggregated prediction result.

## 4. Empirical Analysis

### 4.1. Data Description

In 2020, the annual aquaculture production of larimichthys crocea in China reached 254,000 tons, accounting for 14.5% of the total marine fish aquaculture production in the country. The larimichthys crocea aquaculture industry has become one of the distinctive pillar industries in the eastern Fujian Province.

**Figure 2:**
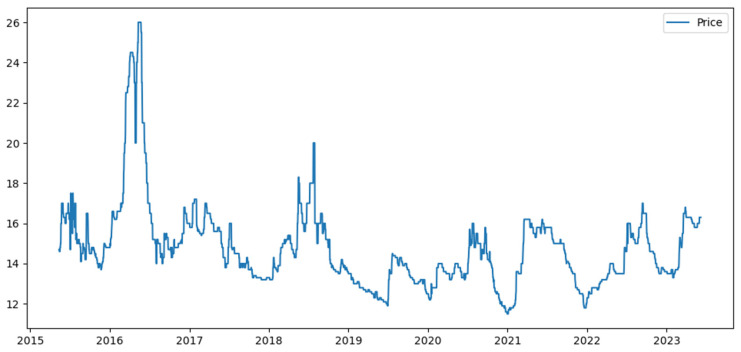
Price trend of Ningde’s larimichthys crocea from 13 May 2015 to 24 May 2023.

This research data came from the WeChat official account platform of “Ningde Larimichthys Crocea”, operated by the Fishery Association of Ningde City, Fujian Province, and selected the daily reference price for sea surface purchase of larimichthys crocea of 400–500 g published by the platform as the research object. The selected time period ranged from 13 May 2015 to 24 May 2023, and missing values were filled using the backward filling method. A total of 2948 daily price data points were collected, as shown in Figure 2. 

Descriptive statistics for the reference purchase prices of larimichthys crocea in the Ningde sea area covering the period from 13 May 2015 to 24 May 2023 are presented in Table 1 (in Chinese Renminbi, RMB). The time series data exhibited right asymmetry and platy kurtosis (non-Gaussian distribution), indicating high volatility and challenging predictability. Therefore, a systematic investigation into forecasting methodologies was warranted to enhance the efficiency of predicting these non-Gaussian time series datasets. The sample data was divided into three subsets. The training set consisted of the first 80% of the price series, totaling 2358 data points; the validation set consisted of the remaining 20% of the series, totaling 590 data points.

### 4.2. Evaluation Indicators

To accurately evaluate the prediction ability of different models, this paper used five indicators to evaluate the models: mean square error (MSE), root mean square error (RMSE), mean absolute error (MAE), absolute percentage error (MAPE), and symmetric mean absolute percentage error (SMAPE). It is crucial to clarify that the mean absolute percentage error (MSE, RMSE, MAE, MAPE, SMAPE) values were computed specifically for the validation sample, and not for the entire dataset. This distinction ensured the accuracy and relevance of the assessment, focusing on the predictive performance within the validation set rather than across the entire dataset.

(6) $$MSE=\frac{1}{N}\sum_{i=1}^{N}\;{\left({\hat{x}}_{t}-x_{t}\right)}^{2}$$

(7) $$RMSE=\sqrt{\frac{1}{N}\sum_{i=1}^{N}\;{\left({\hat{x}}_{t}-x_{t}\right)}^{2}}$$

(8) $$MAE=\frac{1}{N}\sum_{i=0}^{N-1}\left|{\hat{x}}_{t}-x_{t}\right|$$

(9) $$MAPE=\frac{1}{N}\sum_{i=1}^{N}\left|\frac{{\hat{x}}_{t}-x_{t}}{x_{t}}\right|\times 100\%$$

(10) $$SMAPE=\frac{2}{N}\sum_{i=1}^{N}\frac{\left|{\hat{x}}_{t}-x_{t}\right|}{\left|{\hat{x}}_{t}\right|+\left|x_{t}\right|}\times 100\%$$

## 5. Results

### 5.1. Decomposition Result

**Figure 3:**
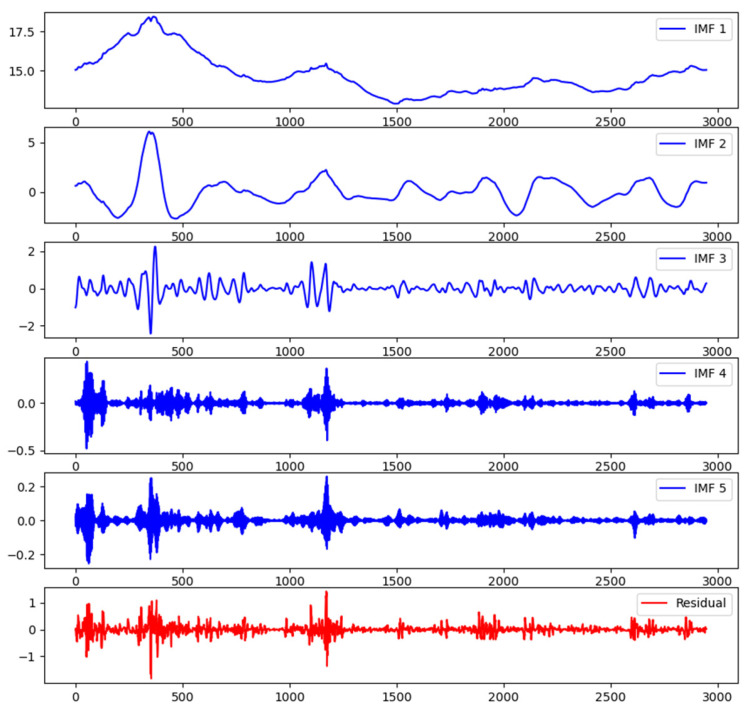
VMD decomposition.

In VMD decomposition, the parameter K represents the number of decomposed modes. A value of K that is either too large or too small can result in excessive noise or loss of information, which adversely affects the accuracy of subsequent predictions. Therefore, when selecting VMD parameters, it is necessary to balance the relationship between the value of K and prediction accuracy. Generally, the optimal value of K can be determined through methods such as cross-validation to ensure that VMD effectively extracts the important oscillatory patterns from the data and establishes accurate models for subsequent data analysis and prediction. After conducting experiments, the final decision was made to set the value of K in VMD decomposition as 5. The decomposed IMF components are shown in the following Figure 3.

**Figure 4:**
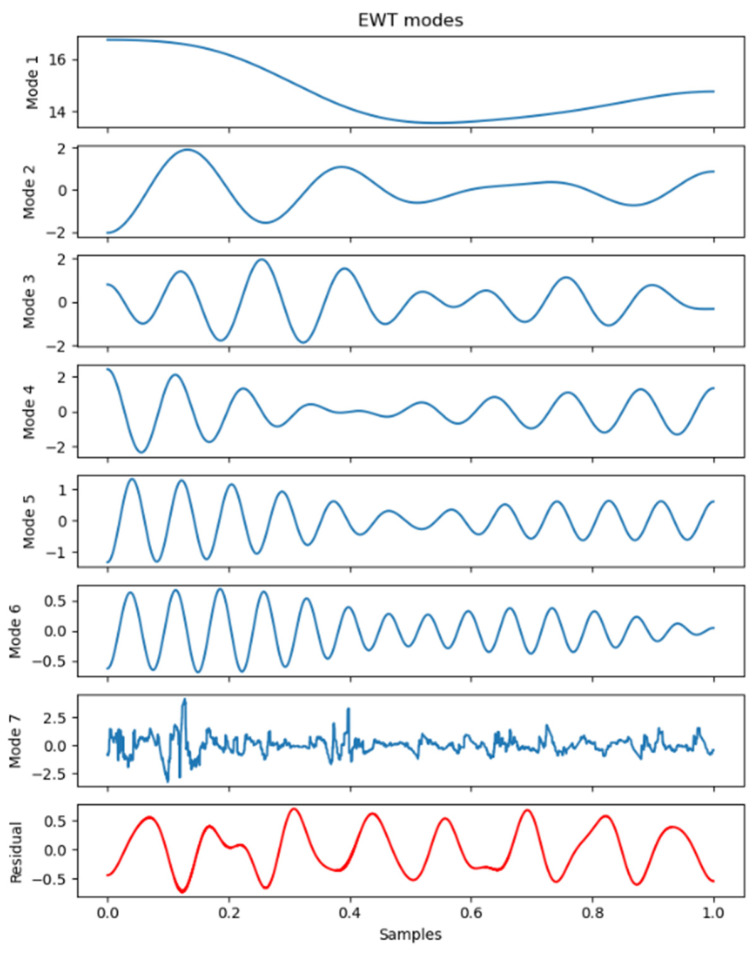
EWT decomposition.

In this study, the EWT (empirical wavelet transform) algorithm was employed to decompose the original sequence. Subsequently, seven empirical mode components (EMCs) were obtained, as shown in the Figure 4. It can be observed that the EWT decomposition results exhibited a characteristic shift from low frequency to high frequency.

**Figure 5:**
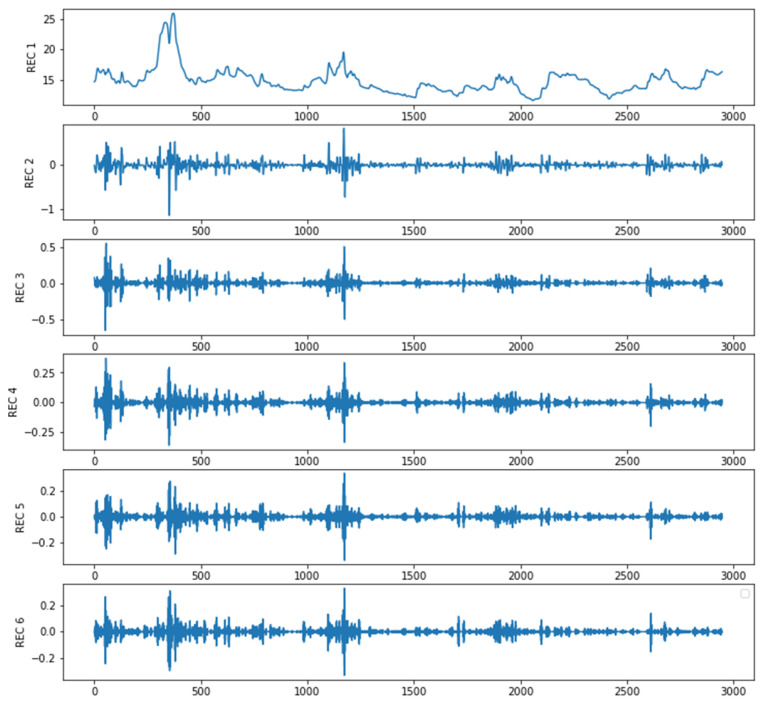
SSA decomposition.

The original sequence was subjected to embedding, decomposition, grouping, and recombination steps through singular spectrum analysis (SSA), resulting in the extraction of six distinct component sequences, as illustrated in Figure 5.

### 5.2. Single-Model Prediction Results

**Figure 6:**
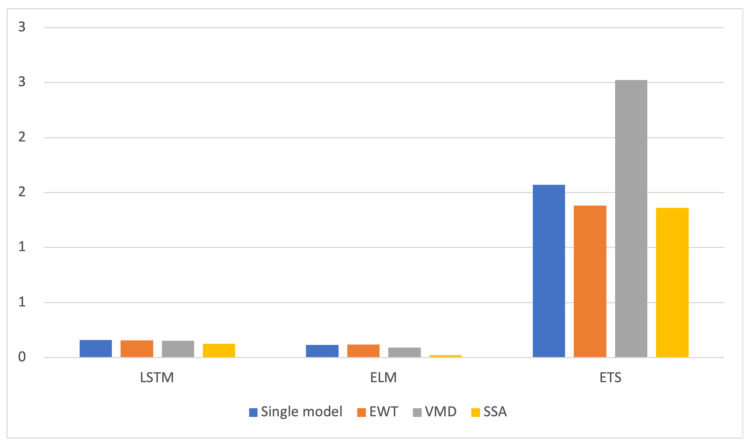
MAPE values for each single model of the sea surface purchase price of greater amberjack (*Lepomis macrocephalus*).

**Figure 7:**
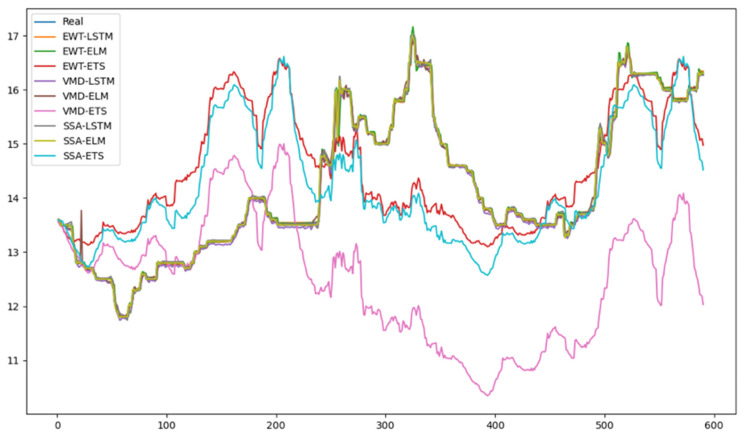
Results of different combinations of prediction models for greater amberjack.

After decomposing the original sequence, each component was subjected to combined predictions using LSTM, ELM, and ETS models. The predictive performance of individual models was evaluated, and the results are presented in Table 2. The corresponding MAPE accuracies for different single models are illustrated in Figure 6. The line chart of prediction results is shown in Figure 7.

Firstly, in most cases, the predictive accuracy of the non-decomposition model was higher than that of the models using EWT, VMD, and SSA decomposition techniques. However, the predictive accuracy of the ETS model based on VMD decomposition was lower than that of other decomposition models. In ETS prediction, errors in the VMD decomposition results were amplified.

Secondly, it can be observed that the predictive accuracy of the models based on LSTM and ELM were higher than that of the ETS model. The main reason for this is that LSTM and ELM are artificial intelligence algorithms that excel in handling information contained in different components after decomposition. Generally speaking, they have stronger adaptability compared to linear models. Prediction models based on artificial intelligence algorithms are more suitable when combined with decomposition techniques. On the other hand, the ETS model is a complex nonlinear prediction model that is more suited for short-term trend forecasting. These findings indicate that the performance of decomposition techniques and prediction models tends to be diverse. Therefore, the combination of models is particularly important for mitigating the risk of model selection.

Regarding decomposition techniques, the predictive model using the SSA decomposition technique yielded better results in predicting the acquisition price of Spanish Mackerel on the sea surface compared to the predictive models using EWT, VMD decomposition, and no decomposition. The MAPE values were reduced by 37.57%, 58.54%, and 42.88%, respectively, indicating that the SSA decomposition technique effectively discovered the hidden factors behind the price fluctuations in this dataset.

### 5.3. Combined Model Prediction Results

This section presents the performance of PSO–CS weight allocation for combined prediction. Table 3 showcases the weight allocation results for the combined prediction of the acquisition price of larimichthys crocea in the sea.

Table 4 and Table 5 present the evaluation of the predicted results and the reduction in prediction error for the PSO–CS weighted combination forecasting of larimichthys crocea’s sea surface purchase price. In most cases, the performance of the PSO–CS weighted combination method was superior to that of the single-model forecasting methods. PSO–CS overcomes the limitations of linear methods by adaptively optimizing weights instead of directly classifying weights based on the performance of individual models, resulting in better performance. Compared to single-model forecasting methods, the PSO–CS approach reduced MSE, RMSE, MAE, MAPE, and SMAPE by 44.43%, 22.69%, 12.21%, 22.88%, and 14.13%, respectively.

However, it exhibited poor performance in the ETS-based forecasting method. This may be due to the larger errors in the ETS during the single-model forecasting process, which hindered the effective utilization of PSO–CS in the weight allocation stage, leading to inferior results. Similarly, the VMD-ETS-based forecasting already yielded significant errors in the single-model forecasting stage, making it challenging for the PSO–CS weighted combination model to demonstrate its performance in the weight allocation stage.

**Figure 8:**
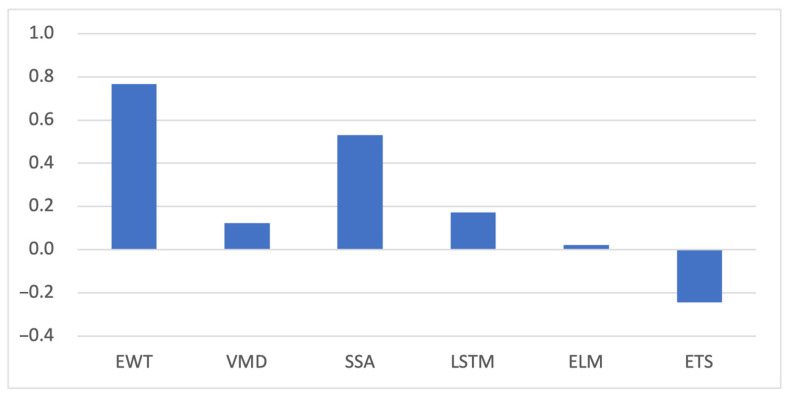
Average error reduction of PSO–CS method relative to single model.

Figure 8 illustrates the reduction in average error of the PSO–CS method compared to single-model predictions. Considering the above, the experimental results effectively demonstrate the robustness of the proposed PSO–CS hybrid model.

### 5.4. Results and Discussion

Understanding the forecast horizon required by decision-makers is crucial for effective decision-making when conducting price predictions. The forecast horizon can vary depending on the application scenario, covering various needs from short-term to long-term. For instance, if decision-makers need to make decisions in the coming months, then price predictions for the next month may be more practically meaningful. Conversely, if decision-makers need to plan for future years strategically, then predictions spanning two years or longer may be more critical.

In this study, we recognized that different decision-makers have varying demands for price predictions over different time horizons. Therefore, this paper chose daily prices with higher volatility as the research focus, as it better captured the factors influencing prices. Furthermore, the ratio of the training set to the validation set was set at 4:1. Our goal was to develop a flexible and customizable intelligent combinatorial model that could generate price predictions over different time horizons according to the needs of decision-makers. We recommend that decision-makers maintain a sample size and forecast horizon ratio of 4:1 when using our model for predictions. This way, whether short-term strategic planning or long-term strategic planning is the goal, our model can provide accurate and reliable prediction results to assist decision-makers in making informed decisions.

The proposed predictive model in this paper may encounter variations in the availability and quality of aquaculture market data across different countries. When conducting cross-national comparisons, particular attention should be paid to the completeness, accuracy, and timeliness of the data. Cultural factors and consumption habits in the seafood market can also differ significantly between countries. For instance, demand levels and price sensitivity for certain marine fish may vary noticeably. Therefore, when conducting cross-national comparisons, it is crucial to consider these cultural and market differences, as different markets may potentially require customized analyses and model adjustments. Additionally, different types of marine fish (such as fish, shellfish, crustaceans, etc.) may exhibit varying data behaviors in the market, necessitating targeted data collection and analysis. Whether the proposed model’s performance in the Chinese market can be extrapolated to other countries’ seafood markets and applied to other types of marine fish products needs to be assessed through a series of validations and tests, possibly employing techniques like cross-validation and model comparisons to evaluate the model’s robustness and applicability. The proposed model in this paper provides some reference value for price prediction in global marine fish markets.

## 6. Conclusions

The prediction of marine fish prices holds significant importance in the fields of agriculture and fisheries. This study investigates the design and application of an intelligent weight allocation method, which offers decision-makers more scientifically and reliably predicted price outcomes. This aids in guiding relevant decision-making and strategic planning processes. It is crucial for decision-makers, producers, and consumers alike. Decision-makers can utilize price forecasts to formulate rational policies and strategies that promote sustainable development and maximize benefits. These aid regulatory bodies in market supervision by maintaining a fair competitive environment. Producers can devise reasonable production plans and supply chain management based on the forecast results, thereby meeting market demand and enhancing efficiency. Consumers can better plan their purchasing behavior through price predictions, avoiding economic burdens resulting from future price increases.

This paper proposes a weight allocation model based on PSO–CS, which effectively integrates multiple decomposition and forecasting models to improve the accuracy and stability of price prediction in marine fish. By utilizing daily purchase prices of larimichthys crocea in Ningde City, Fujian Province as data, several conclusions were derived regarding the sample interval. Firstly, the decomposed ensemble prediction model significantly improved the predictive performance compared to direct prediction models. Secondly, the accuracy of the SSA-ELM prediction model was 46.33% lower than the other three models on average, which was better than the other individual models. Thirdly, the combination prediction model based on PSO–CS weight allocation exhibited significantly superior performance compared to single-model predictions. The limitations of this study lie in the potential performance of the model in specific contexts, which may be favorable, but its applicability in other market environments or periods requires further verification and exploration. Additionally, the seafood market is influenced by various external factors such as climate change and policy adjustments, which could impact the model’s predictive outcomes and necessitate consideration and control measures.

In conclusion, the marine fish price prediction framework based on weight allocation intelligent combinatorial modelling holds significant importance in the agricultural and fisheries sectors. Our research provides a scientific basis for decision-making and business operations in related fields, offering beneficial insights for future research and practical applications.

## Figures and Tables

**Table 1:** Descriptive statistics of the reference price of the sea surface purchase of Ningde’s larimichthys crocea from 13 May 2015 to 24 May 2023. RMB.

Norm	Average	Maximum	Minimum	Upper Quartile	Standard Deviation	Skewness	Kurtosis
	14.7436	26.0000	11.5000	14.5000	2.1167	2.3174	8.3753

**Table 2:** Assessment of the results of the single-model prediction of the sea surface purchase price of greater amberjack (*Lepomis macrocephalus*).

Models		MSE	RMSE	MAE	MAPE	SMAPE
LSTM	Single model	0.0256	0.1599	0.0693	0.4846	0.6528
	EWT	0.0246	0.1569	0.0658	0.4525	0.6173
	VMD	0.0231	0.1518	0.0748	0.5217	0.6275
	SSA	0.0159	0.1259	0.0428	0.2989	0.4840
ELM	Single model	0.0136	0.1165	0.0479	0.3282	0.5712
	EWT	0.0137	0.1170	0.0551	0.3770	0.6132
	VMD	0.0081	0.0902	0.0481	0.3307	0.4889
	SSA	0.0006	0.0240	0.0113	0.0775	0.3314
ETS	Single model	2.4673	1.5708	1.1937	8.8844	7.7578
	EWT	1.9050	1.3802	1.1124	7.5960	7.6910
	VMD	6.3686	2.5236	2.0953	17.5228	15.6253
	SSA	1.8503	1.3603	1.0969	7.6466	7.6380
Ave		1.0597	0.6481	0.4928	3.7101	3.5915

**Table 3:** Results of the prediction weight allocation for the combination of the sea surface purchase price of greater amberjack.

Models	Weight 1	Weight 2	Weight 3	Mini Fitness
EWT	0.97762	0.017449	0.054719	5.4956
VMD	0.45978	0.20007	0.39683	4.7631
SSA	0.3202	0.56079	0.16737	5.5289
LSTM	0.27499	0.69521	0.081055	5.5193
ELM	0.54832	0.31219	0.19195	5.5163
ETS	0.13105	0.90177	0.022209	8.0085

**Table 4:** Assessment of the results of the forecasting of the sea level purchase price combinations for greater amberjack.

Models	MSE	RMSE	MAE	MAPE	SMAPE
based on EWT	0.01857427	0.13628745	0.09340442	0.65263831	0.818293917
based on VMD	0.92698336	0.96279975	0.79625986	5.37405448	5.594460685
based on SSA	0.04924591	0.22191419	0.1785835	1.2566242	1.327562983
based on LSTM	0.00845136	0.09193128	0.05254116	0.36355915	0.57555956
based on ELM	0.00692352	0.08320768	0.03967529	0.27210333	0.494659372
based on ETS	5.16845769	2.27342422	1.91025014	12.9420506	14.04080338
Ave	1.02977268	0.62826076	0.51178573	3.47683835	3.80855665

**Table 5:** Reduction rate of assessment error for the combination of forecasting results of the sea surface purchase price of greater amberjack (*Lepomis macrocephalus*).

Models	MSE	RMSE	MAE	MAPE	SMAPE
based on EWT	0.97132576	0.75282352	0.77280464	0.76762124	0.72438143
based on VMD	0.56545996	−0.0443994	−0.0768843	0.12261747	−0.0041503
based on SSA	0.92085782	0.55914784	0.5345503	0.53012071	0.52806995
based on LSTM	0.62066388	0.38158246	0.16872412	0.17270049	−0.0004921
based on ELM	0.22984798	0.04248147	0.02329428	0.02244412	−0.0369583
based on ETS	−0.6419242	−0.3304881	−0.3896967	−0.2429374	−0.36305
Ave	0.44437186	0.22685796	0.17213207	0.2287611	0.141300095

## Data Availability

The datasets generated during the current study are available from the corresponding author on reasonable request. The data in this study came from the Fisheries Association of Ningde City, Fujian Province, China. The original contributions presented in the study are included in the article, further inquiries can be directed to the corresponding authors.
